# Supplementary material for: Mechanisms governing the pioneering and redistribution capabilities of the non-classical pioneer PU.1
Source: Nat Commun. 2020 Jan 21;11:402. doi: 10.1038/s41467-019-13960-2 (PMC6972792; doi:10.1038/s41467-019-13960-2)
Supplement: Supplementary file 7 — Source data [file 41467_2019_13960_MOESM7_ESM.zip › Source_Data/FigureS2/FigureS2B_MotifScanOutput/KmeansCluster5/homerResults/motif23.similar.html]

motif23

## Information for motif23

C
T
A
G
C
T
A
G
A
C
T
G
C
T
G
A
C
T
G
A
T
C
A
G
A
G
T
C
G
A
T
C
G
A
C
T
A
G
T
C
C
G
T
A
A
C
T
G
C
T
A
G
C
T
G
A
  
Reverse Opposite:  

A
G
C
T
G
A
T
C
A
G
T
C
C
G
A
T
C
T
A
G
C
T
G
A
C
A
T
G
T
C
A
G
A
G
T
C
A
G
C
T
G
A
C
T
G
T
A
C
A
G
T
C
G
A
T
C
  

|  |  |
| --- | --- |
| p-value: | 1e-43 |
| log p-value: | -1.005e+02 |
| Information Content per bp: | 1.719 |
| Number of Target Sequences with motif | 123.0 |
| Percentage of Target Sequences with motif | 3.09% |
| Number of Background Sequences with motif | 227.6 |
| Percentage of Background Sequences with motif | 0.53% |
| Average Position of motif in Targets | 77.3 +/- 70.4bp |
| Average Position of motif in Background | 103.9 +/- 67.7bp |
| Strand Bias (log2 ratio + to - strand density) | 1.0 |
| Multiplicity (# of sites on avg that occur together) | 1.00 |
| Motif File: | file (matrix) reverse opposite |

### Similar de novo motifs found

|  |  |  |  |  |  |  |  |
| --- | --- | --- | --- | --- | --- | --- | --- |
| Rank | Match Score | Redundant Motif | P-value | log P-value | % of Targets | % of Background | Motif file |
| 1 | 0.886 | G A C T G T A C A G T C A G C T A C T G G T C A C A T G C T A G A G T C A G T C C G A T T A G C A G T C | 1e-35 | -81.816305 | 2.24% | 0.33% | motif file (matrix) |
| 2 | 0.899 | C A T G T A C G T A C G C G T A C T A G C T A G G T A C G A T C A G C T A G T C C T G A C T A G | 1e-35 | -81.592585 | 2.81% | 0.56% | motif file (matrix) |
| 3 | 0.782 | A G C T G A T C A T G C A G C T A C T G C G T A A C T G T C A G G A T C A G T C A C G T | 1e-29 | -68.365672 | 3.19% | 0.85% | motif file (matrix) |
